# Atlanta Academy of Medicine

**Published:** 1872-01

**Authors:** 


### ATLANTA ACADEMY OF MEDICINE.

REPORTED BY JAS. B. BAIRD, M.D.

October 26, 1871.

The President, Dr. Logan, in the chair.

The President announced that the discussion of the question proposed by the Section on Hygiene was in order; whereupon the discussion was opened by Dr. J. B. Baird, who reminded the Academy that the question for discussion was the Hygiene of Gestation. He then explained that the Section had determined to divide the main question into twelve divisions, and that these divisions had been distributed to three members of the Section, who would respectively present the part selected, and any remarks they might see fit therewith. Furthermore, that they had prepared what they had to say, and would present it in writing—thereby saving time, opening the discussion more thoroughly, etc. He then briefly called the attention of the Academy to the prevention of nausea during gestation, referring to its cause, results, and treatment.

Dr. Jno. Stainback Wilson followed, reviewing minutely the following subjects, divisions of the main question, viz: First, on the conditions favorable to its inception; second, on food; third, on clothing; fourth, on ventilation and insolation; fifth, on labor and inactivity. The Doctor entered at some length into the consideration of each of the above questions. (Published in December number of the JOURNAL.)

Dr. Cumming then concluded by calling attention to the remaining divisions of the question before the Academy. The Doctor lucidly and pointedly considered the following: First, on nervous exhaustion and its influence; second, on lactation during the progress of gestation; third, on the relation of hygiene of gestation to immediately-succeeding lactation; fourth, on the influence of disease on gestation, and the importance of prevention; fifth, on the special hygiene of a first pregnancy.

The question was then declared to be before the meeting for general debate.

Dr. Bozeman.—Would inquire if any one present has had any experience among savages, and if they suffered as much during gestation and parturition as the more civilized nations?

Dr. Cumming.—Had never lived among savages, but has learned from the books that they suffer little. Indian women are said to be nearly exempt—usually bring forth their young on the march. Had friends in Amoy, China, who had lived among the Dyaks of Borneo, where the women suffered very little, giving birth to children in the woods, etc. Among our own population, illegitimate births frequently occur, privately, with little or no hemorrhage. Cases are recorded where the child has suddenly dropped from the mother.

Dr. Wilson.—The Indian women, after parturition, are careful to wash themselves, and this may account for the absence of signs of hemorrhage. Has seen several women accomplish the act of parturition with two or three *painless* contractions. Thinks, in the great majority of cases, where women are well-formed, labor is attended with no difficulty.

Dr. Bozeman.—Thinks that labor is easier with the negro than it is in the so-called highly civilized through hygiene; or, in other words, a simple mode of living is the basis of easy parturition.

Dr. Cumming.—It has been estimated that the force of the abdominal muscles is to the uterus as five or eight to one. The contractions of the uterus produce pain. In well-developed women, most of the work of parturition is done by the abdominal muscles. Most women, in the better classes of society, have thin abdominal muscles—have never been developed. This is one reason for cultivating in women a high degree of muscular power.

Dr. Bozeman.—Will add a word to the same purport. A primitive condition of life is favorable to a painless parturition. Has seen cases, among the Mexican Indians, where a woman would give birth to a child one day, and be engaged at her regular work on the day following. This may be explained from the fact, that owing to their mode of life, the weakly women die off early; while we do raise our weakly women. Referring to the negro, thinks all laboring, well-fed classes bear children; but he does not think the negro, in difficult labors, can sustain as much the white race. They succumb sooner—have not the same resisting power. Has only met with two cases of ruptured uterus in his practice—both in negro women.

Dr. Wells.—Has traveled in the West. Thinks the Indians suffer less in child-bearing, than the whites. Agrees with Dr. Bozeman in his reference to the laboring classes. The women of the South are raised like hot-house plants. Many ladies have a much better developed mind than body. These suffer more than those raised more roughly. In his experience, the negro's labor is generally shorter than the white's; but, in difficult cases, they succumb sooner. The head of the negro is not so round as the head of the white child, especially the German. Instrumental labors are more common among the Germans than with us—not on account of their greater advancement in the science, but that the heads of their children are larger and rounder. The negro's head is long and narrow. Thinks, in civilized life, women are raised that, in savage life, would not reach maturity. Thinks the disproportion between the head and pelvis is generally the cause of delay, not the want of expulsive force.

Dr. Wilson.—In reply, we all admit that there must be a well-formed pelvis. Have had cases in which the labor was difficult, and subsequent parturitions, after following the course laid down in my paper relative to the hygienic management of gestation, were easy. Does not agree with Dr. Cumming, that pain is caused by contraction of the uterus, but thinks it is caused mainly by pressure upon and stretching of the soft parts, and morbid irritability.

Dr. J. G. Westmoreland.—How do you account for after-pains?

Dr. Wilson.—A woman that lives right does not have after-pains, if properly treated after delivery.

Dr. Logan.—What is proper treatment?

Dr. Wilson.—Remove clots, and inject cold water into the vagina.

Dr. Harden.—Thinks Dr. Wilson, in his essay, attaches too much importance to exercise. It must be very moderate. Remembers his experience on two adjoining plantations: on one, there was no provision made for indulgence to pregnant women, and there was no increase of population; on the other, every requisite provision was made for pregnant women, and consequently rapid increase in population. Thinks abortion is frequently brought about by exercise — ascending stairs, etc.

Dr. J. S. Wilson.—Deprecates violent, but urges moderate, exercise.

Dr. Bozeman.—Thinks the point made by Dr. Harden a good one. Regular daily exercise is different from slave labor. In his experience, pregnant women were taken from the field, and furnished lighter employment. Thinks they ought to be withdrawn from field labor. Thinks the subject for our discussion has been so well presented, that he wishes to criticise a little. Thinks pregnant women, even if suffering with sick stomach, should eat; if they vomit, eat again. They should be encouraged to eat. In latter months, it is urged that women must not eat too much. Thinks they must not eat for two, but may require more than enough for one. In regard to the regulation of the bowels, it has always been a hobby of the profession to regulate the bowels of the country. Leave something for the bowels to do for themselves. Does not condemn all attention to the bowels, but one or two actions a day may be detrimental to women approaching the great work of labor. Thinks he can recall two or three cases in which "regulation of bowels" complicated labor. The indiscriminate use of seidlitz powders and cream of tartar is productive of much harm.

Dr. Wilson.—I combatted the popular notion that a pregnant woman should eat for two, but think she should eat as much as she can digest. Agrees fully with Dr. Bozeman in regard to regulating bowels. Is an advocate for withholding all laxative medicines in pregnancy. The bowels should be regulated by food, by a judicious diet. Thinks purgatives are as much abused now as the lancet was formerly. Regards purgatives, administered immediately after delivery, injurious.

Dr. J. G. Westmoreland.—I think no condition demands purgatives more than that of a female the day after delivery. We have a febrile condition, which may be extended. We have, on the second or third day, a fever setting up—a prostration following, if fever is not subdued; no means so certain as a purgative. This is the time. I administer the purgative.

Dr. Bozeman.—Does Dr. Westmoreland refer to a diseased or a healthy woman?

Dr. J. G. Westmoreland.—I give it any way, to prevent the fever.

Dr. Boring.—I rise to a point of order. We are discussing gestation, not post-partum conditions.

Dr. Westmoreland.—Dr. Cumming's paper was largely devoted to discussing lactation; that is post-partum.

Dr. Cumming explained that his allusion to lactation was in reference to the effect of the hygienic condition of the female during gestation upon the subsequent and immediately-succeeding lactation.

The President sustained the point of order made by Dr. Boring.

Dr. Raushenberg.—If I understand Dr. Wilson, he says, by hygienic means during gestation, labor may be shortened and made painless. Is the most normal process of labor the one which requires the least space of time, and connected with the least amount of pain? As labor is a mechanical process, occupying several stages—involving the necessity of dilatation of the os, of the gradual passage of the fœtus through the pelvis, the gradual dilatation of the external parts, etc.—it must necessarily occupy, under all and the most favorable circumstances, and for the very safety of the female, a number of hours; and I am certain that the very first woman who ever had a child occupied a good deal of time and suffered a good deal of pain, and that no course of hygiene will ever make labor painless, or reduce its duration.

Dr. Wilson.—I dislike to speak so frequently, but must defend my position. I think labor is a natural, physiological act. Then, why should it be attended with pain? We can empty our bowels and our bladders with pleasure; why not the uterus? Thinks the pains are due to the rigidity of the perineum and other parts. A woman carried through a system of hygiene will have an easy labor. Have seen it tried. If everything goes on *pari passu*—if delivery occurs in five minutes—all right.

Dr. Boring.—I do not think the shortest labors the safest. Can recall two cases of recent occurrence: one was not in labor ten minutes, and has not done so well since as another who was in labor several hours.

Dr. Raushenberg.—Does not think Indians, or any savages, are delivered walking. The os may dilate, but they must stop to be delivered.

Dr. Wilson.—O, yes, they stop a little while, but soon overtake the party when on the march.

Dr. Raushenberg.—Will ask Dr. Wilson what is the normal time for a labor?

Dr. Wilson.—If the parts are prepared, and everything proceeds *pari passu*, it can be accomplished in five minutes.

Dr. Raushenberg.—Do you think the mouth of the womb can dilate in five minutes?

Dr. Wilson.—I maintain that a woman can be put in a condition, by attention to hygiene, so that delivery will take place in three hours; whereas, by neglecting these measures, six would be required.

Dr. Raushenberg.—I have, as a deviation from normal labor, seen once a case where a market-woman had a child in a very short time after conveying a basket of vegetables to to town, and was able to carry it home in her basket on the same day. But these are not normal cases, and my experience has taught me, that those cases of delivery which occupy the least time are connected with most danger to the mother, from hemorrhage and other puerperal diseases.

Dr. Bozeman.—What is the experience of the gentlemen present with the use of chloroform in labor?

Dr. Cumming held that this subject did not relate to hygiene.

The point made by Dr. Cumming was sustained by the President.

---

*Hygiene of Gestation. By Jas. B. Baird, M.D., Chairman of the Section on Hygiene, being part of a Report to the Atlanta Academy of Medicine.*

It will be recollected that the question proposed by the Section on Hygiene, for discussion, was the hygiene of gestation. This subject is very comprehensive, and full of interest and importance to the general practitioner. It was thought by the Section that its consideration would be invested with more interest, and that the subject would be investigated more thoroughly and more systematically, by dividing the question into several parts, and these divisions be presented by different members of the Section.

It was further determined to present any views held by the Section in writing, as, by so doing, time would be economized, more time would be given to the study of the subject, a better basis furnished for remarks by the members of the Academy, and, by a division of the labor, a more complete analysis of the question would be obtained. Accordingly, the question was divided into twelve parts, as follows:

1. On the conditions favorable to its inception.
2. On food.
3. On clothing.
4. On ventilation and insolation.
5. On labor and inactivity.
6. On nervous exhaustion, and its influence.
7. On lactation during the progress of gestation.
8. On the prevention of nausea.
9. On the relations of hygiene to immediately-succeeding lactation.

10. On the influence of disease on gestation, and the importance of prevention.

11. On the special hygiene of a first pregnancy.

12. On the prevention of abortion.

These have been distributed among three members of the Section, who propose to offer a few observations upon the divisions selected, giving a short *résumé* of each, together with any original ideas which they may entertain, or have derived from actual experience in this department of practical medicine. These papers are not intended to be exhaustive—and it is earnestly hoped that they will not prove exhausting—but, as before stated, simply to present the subject in a more tangible form, with a proper classification of its different departments.

I propose to call the attention of the Academy, very briefly, to the eighth of the above list, viz: The prevention of nausea.

That nausea, with more or less vomiting, is often one of the most troublesome symptoms that presents itself in the female during gestation, all will admit; hence, the consideration of its cause and treatment is of no secondary importance. It is true that it rarely proves fatal, but the discomfort and exhaustion incident to it are very distressing, and in many cases it is followed by extreme emaciation.

It is proper, first, to inquire as to the cause of this nausea. Cazeaux says: "When it occurs near term, we may justly attribute it to pressure—to the mechanical constraint which the uterus, whose fundus reaches the epigastric region, exercises upon the stomach; but in the early stages, it is much more difficult to explain it, unless we content ourselves by referring it to the numerous sympathies existing between the uterus and the stomach—sympathies so intimate that they are manifested in certain women at every menstrual period, and even in nearly all those afflicted with a disease of the womb." Dr. Bennett thinks that this obstinate vomiting is almost always caused by "inflammatory ulcerations of the neck of the womb."

Can nausea and vomiting be accounted for, in the early stages of pregnancy, by pressure upon the abdominal organs, produced by the rigidity of the abdominal walls, which fail readily to accommodate themselves to the changed relations of the uterus?

Are primiparæ more subject to this distressing complication than multiparæ? It is generally believed that they are; and if this popular opinion was sustained by statistics, it would be an argument in favor of the theory suggested by the above question, as their abdominal muscles are less yielding than those of women who have borne children. But this is by no means settled. If it is generally so, there are certainly many exceptions; even some women escape during their first pregnancy, but are attacked in subsequent pregnancies. In those cases where nausea and vomiting are present, it varies much in severity; in some instances it is attended with little or no suffering, while on the other hand it frequently occasions much epigastric pain. The danger, even though the vomiting be excessive, should not be over-estimated; for though this symptom be very prominent, and emaciation proceed to an alarming extent, yet the female may bring forth a large, healthy child, and suffer no permanent injurious effect herself. It is claimed that the violent efforts incident to vomiting may produce abortion, but this must be extremely rare. The diagnosis is usually sufficiently easy. It is not likely to be mistaken for vomiting produced by other causes.

But the great point of interest is the treatment. Unfortunrtely, we have no specific to offer, and the great variety of medicines which have been proposed for this affection, from time to time, is evidence of the uncertainty of drugs in its control. It is unnecessary to mention here the long list of so-called remedies that have been used with advantage in some instances, and which have entirely disappointed the expectations of the practitioner in other and apparently similar cases. In some cases, the subnitrate of bismuth has been used with marked relief; in others, the aromatic infusions, different preparations of mercury, alkalies, alcoholic liquors, topical, stimulating and anodyne applications over the epigastrium, and more recently the oxalate of cereum and carbolic acid, have been extolled by many practitioners. General blood-letting formerly had many advocates, and it is still resorted to by some.

This list might be extended to almost every article in the *Materia Medica*, but it is unnecessary to pursue it further.

Cazeaux, in his work on obstetrics, reports a case which yielded promptly to the application of belladonna to the os uteri, after resisting every other mode of treatment. One of the most successful plans with which I am acquainted, and one eminently *hygienic*, is to require the sufferer to take something on her stomach before arising in the morning—a cup of coffee, a glass of milk and lime-water, or a milk punch—consulting, of course, the preference of the patient in the selection of the article prescribed.

A physician* of eminence in this city, and of an extensive experience, recommends, in troublesome cases, that the *morning meal* be taken in bed, and that the patient remain in bed an hour or two after breakfasting.

If, as has been suggested, the unyielding nature of the abdominal walls, in the early stages of pregnancy, exert an agency in the production of this distressing complication, probably small doses of chloroform by the stomach, frequently repeated, would be palliative, if not curative.

In conclusion I will remark that the generally limited duration of the symptom, and in many cases its sudden cessation, render it difficult to ascribe to any mode of treatment its exact value.

---

*Hygiene of Gestation. By Wm. Henry Cumming, M.D., being part of a report to the Atlanta Academy of Medicine from the Section on Hygiene.*

The work of reproduction is a function added to those of individual life. Our watches may be good time-pieces, and, thus, most useful articles, without possessing the power, hypothetically attributed to them by Archdeacon Paley, of constructing other watches similar to themselves. But it would seem to be a mistake for a watch, not yet perfect in its own structure, to be charged with the preparation of other watches; neither should an immature and physically imperfect woman undertake the work of reproduction. In like manner,

*Dr. William Henry Cumming, Atlanta, Georgia.

(to continue Paley's analogy,) if the motive power of a watch were insufficient, or barely sufficient, to maintain its time-keeping parts in motion, it would certainly be unwise to add to its labor by the addition of the watchmaking apparatus. In other words, generation, especially that part of it devolving upon women, implies the consumption of vital force, and, therefore, demands extraordinary supplies for its proper performance. In immediate sequence come the three acts of the drama—ovulation, gestation and lactation. The first function is known to be closely dependent on the general energy of the system, and usually indicates this connection by positive and apparent failure whenever the bodily powers decline. None but vigorous women ovulate well. The function is, in nearly all others, irregular, painful or defective; and so soon as, in such cases, the general health and vigor are regained, the work of ovulation is again well performed. In this case, the indications are so plain that there is no danger of mistake. In the next stage, gestation, the connection exists, though not so readily observed. In the one case, the cycle is one month long; in the other, eight times longer. Or rather, as lactation usually follows, the cycle may be considered as lasting two full years, and opportunities of observation and careful comparison are, therefore, comparatively distant. Yet we may not doubt that the proper performance of the work of gestation is as closely connected with the healthful action of the system as is that of ovulation. By extending our observations to the third stage of the generative process, we may greatly add to the breadth of our induction. We see, not only in the case of women, but even more apparently and obviously in the case of domestic animals, that lactation, to be fully and efficiently performed, requires a great amount of general vigor, and that its demands for large supplies of food must not be refused.

It were very unlikely, then, that the function lying midway in the entire series, the process by which the germ, at first microscopic in its dimensions, attains the size and weight of an infant at term, should be equally well performed by a feeble and by a vigorous woman. But we are not left to rely on such inferences alone. Direct and long-continued observation, in many countries of the earth, and extending over long ages of time, clearly shows the difference between the process of gestation in feeble and in vigorous persons. The women in nomadic tribes, on our own continent and in Asia, have often been cited as instances of the successful performance of this function; but we need not leave the abodes of civilization to find many most forcible illustrations.

### ON THE RELATIONS OF THE HYGIENE OF GESTATION TO THE IMMEDIATELY-SUCCEEDING PARTURITION AND LACTATION.

Scarcely any fact is more frequently observed than the difference existing between women in this, as well as to the sufferings and losses connected with parturition. In the most favored cases there is very little suffering, and no hemorrhage worthy of notice. The mother, strong and cheerful, enters amid most favorable conditions, upon the longer and more exhausting work of lactation. To her the season of pregnancy has been a season of abundant nutritions, resulting in bodily vigor, rich blood and considerable adipose accumulations. She has thus secured several of the most important conditions of the successful performance of the immediately-succeeding and long-continued function.

But, on the other hand, we observe women with whom the season of gestation is a season of nausea, of vomiting, of indigestion, of nervous disturbance and suffering, of sleeplessness, resulting in impoverishment of the blood, (amounting, in fact, often to positive anemia,) loss of strength, emaciation, nervous depression and gloomy forebodings of impending ill. We find them inert, secluded, gloomy, seldom leaving their beds and chambers, and hypochondriacally watching every symptom with fear. This weakness, anemia and alarm are most unfavorable to successful parturition, nor is the recumbent posture, so constantly maintained, without its injurious influence upon the position of the child, and, consequently, on the mechanism of labor. Both the involuntary muscles of the womb and the voluntary muscles of the abdomen have lost their tone and power; their movements are less accurately co-ordinated, and, consequently, much less efficient for the expulsion of the contents of the womb. The blood, already deficient in quality, is greatly reduced in quantity by excessive and uncontrollable hemorrhages, and the mother, almost more dead than alive, is evidently unfit even to attempt to nourish still farther the child that has already cost her so dear.

Nor must we omit to notice the probable connection between this anemic condition of the mother, whether preceding or following parturition, and that painful and distressing affection, "phlegmatia alba dolens." Nor must we overlook the necessity of bodily vigor in resisting altogether, or in successfully passing through that scourge of lying-in hospitals, puerperal fever. Nor must we shut our eyes to the fact that puerperal mania seems closely connected with anemic conditions. In fact, a good state of the body is invaluable to any one who may be called to pass this ordeal of parturition.

How seriously does debility, however arising, hinder the work of lactation, diminishing the nutritive value of the milk and rendering the mother unable to withstand the drain which this function makes upon the bodily energies. The child becomes a source of anxiety and alarm. These still farther injure the quality of the milk. The unceasing demands of a growing child upon an already overtaxed system reduces still farther the mother's strength, and, each thus acting most unfavorably, and even injuriously, upon the other, the relation is hurtful to both and has to be dissolved for the good of mother and child.

### ON THE INFLUENCE OF NERVOUS EXHAUSTION OR DEPRESSION.

There are many conditions which may produce this injurious result. Some of these are irremediable. The loss of husband or child, or other object of earnest love, the loss of property, of reputation, of employment, serious and long-continued illness in the household, war, pestilence, siege, battle, have ever borne with crushing weight upon the pregnant, and the nursing mother. "Woe to them that are with child and to them that give suck in those days," was the exclamation of our compassionate Redeemer as, with prophetic vision, he beheld the calamities so soon to fall upon the Jewish nation. Yet, while we may not hope to remove *these* causes of evil, the consideration of their effects may be most useful. There are *many* influences, tending in the same direction, that are more or less under our control, and a knowledge of their injurious effects should lead us to labor for their removal. We should carefully husband the strength of the pregnant, and advise them of the danger to their offspring of any unnecessary exposure amid scenes of suffering. The duty of attendance upon the seriously sick should not be imposed upon, or assumed by such a person, unless in cases of real necessity. And where trouble has come, the sufferer herself, and her friends also, should be warned of the danger from its excess or too long continuance.

And here, let me refer to one cause of nervous exhaustion which is, or ought to be, most carefully avoided. Many young women form the habit of devoting a large part of their waking hours and, in many cases, the hours that should be devoted to sleep, to works of fiction, exciting in themselves, and still more injurious because of the long confinement the habit involves. It is not an exaggeration to say that many young women spend thus from six to twelve hours daily. There are not many women, perhaps, who suffer from nervous exhaustion caused by excessive study; but there are other occupations and employments that frequently produce the same result. Teachers who are earnest in their work, and serving-women who earn their living by the needle, are often completely exhausted by their daily tasks. The nervous energy thus, by an act of the will, directed to the brain, is diverted from the functions of organic life. Digestion becomes tedious and imperfect, assimilation fails, languor marks and characterizes the conduct of the various organs, and thus there is a general decline of the processes which tend to strengthen and build up the life of the mother and of the child. Nowhere are these results more readily seen than among the operatives in factories, and those engaged in shoe-making and other sedentary occupations.

Under the system of African slavery in this country, great care was taken to remove pregnant women from field-labor, and the good results of such a course were evident. If, under the present system of labor, the women of that race shall be compelled to steady work during the entire term of gestation, the results cannot fail to show themselves.

THE INFLUENCE OF DISEASE UPON GESTATION.

Having already adverted to the importance of vigor to successful gestation, it is scarcely necessary to remark that all bodily disorders must be more or less unfavorable. Perfect health is to be desired, and all contagious or endemic diseases should be avoided. A pregnant woman should, if possible, be removed from a malarial region, lest remittent or intermittent fevers should endanger her life or diminish her strength. And yet we often observe that women will pass the season of gestation in a malarial region, which they expect to quit the next summer on account of the then-born infant. Is it likely that the child is safer in one season than in the other? Would it not be better to remove the *fœtus* from such influences?

The various eruptive fevers are specially dangerous to pregnant women, and greatly hinder the work of gestation. Abortions are frequently produced; and it is a certain fact, clearly attested by many cases of variola and of syphilis, that a child may have these diseases in the womb, and yet survive.

The apparently well-attested instances of a mother's contracting secondary or constitutional syphilis from the fœtus in the womb, clearly indicate the closeness of the vital relation existing between them, and may well prepare us to believe that other constitutional affections may thus be communicated indirectly from the husband to the wife. The process would seem to be that the poisoned germ, infinitesimal as it is, multiplies within itself the original poison to such an extent as to infect its own blood, aud then that of the mother. The facts already known concerning primary syphilis, variola and vaccinia, accord well with this view. So far as the blood is concerned, mother and child are one individual, and one cannot suffer without damaging the other.

The field of pathological observation opened by these facts must not be suffered to tempt us to wander from our prescribed path on this occasion. We must confine ourselves this evening to hygiene.

A practical inference, however, from these facts is, that pregnant women should avoid all that tends to injure the quality of the blood. They should not frequent crowded, unventilated rooms, where the air is fetid with animal exhalations. If they are nurses in hospitals, they should, at least for the time, abandon that employment. If the pregnant wife has a husband far advanced in tubercular consumption, she should not occupy his bed nor even his bedchamber. A regard for the welfare of her unborn child should control her conduct in this matter. If the child were born she would not dare to keep it in such an atmosphere. Why may the fœtus be exposed to the same influences and yet escape injury?

ON THE SPECIAL HYGIENE OF A FIRST PREGNANCY.

As the fact of pregnancy is not usually known until some weeks after its commencement, and as injury may occur at a very early stage, care should be taken *from the very first* to avoid all conduct that may result in harm. The vivacious bride, excited by the various objects of interest met with on her marriage tour, is apt to forget that prudence requires her to conduct herself with moderation. The ascent of lofty towers should be interdicted, and sight-seeing should not be carried to exhaustion. Sea-voyages, with their nausea and vomiting, continued sometimes for many days, have often produced abortions, and the danger should be remembered. Dancing is not without its perils, and is ordinarily forbidden by medical advisers. Among women who labor for their livelihood, heavy effort should be avoided. The lifting of large tubs full of water or clothes is risky. Long-continued standing is injurious, and the steady, continuous use of a treadle-moved sewing-machine has occasioned frequent miscarriages. The development of the mammary glands should be permitted, unhindered by the usual compressions. At the later stages the nipples should be hardened by the use of tannin, or other similar application. Exercise should be methodical and regular, not occasional and excessive.

Care should be taken that the child should not become excessively large, and thus increase the sufferings of the first parturition. This moderation should be secured, not by starvation of the mother, but by a sufficiency of good food and active exercise during the entire period of gestation. The sense of shame so common among women pregnant for the first time should not be suffered to confine the expectant mother within doors, but out-of-door exercise should be continued to the very last.

